# Defining growth in small pulmonary nodules using volumetry: results from a “coffee-break” CT study and implications for current nodule management guidelines

**DOI:** 10.1007/s00330-021-08302-0

**Published:** 2021-09-27

**Authors:** Emily C. Bartlett, Samuel V. Kemp, Bhavin Rawal, Anand Devaraj

**Affiliations:** 1 Department of Radiology, Royal Brompton Hospital, Sydney Street, London, SW3 6NP UK; 2 National Heart and Lung Institute, Imperial College, London, SW3 6LY UK; 3 Department of Respiratory Medicine, Royal Brompton Hospital, Sydney Street, London, SW3 6NP UK

**Keywords:** Multiple pulmonary nodules, Lung neoplasms, Growth, Cancer screening

## Abstract

**Objectives:**

An increase in lung nodule volume on serial CT may represent true growth or measurement variation. In nodule guidelines, a 25% increase in nodule volume is frequently used to determine that growth has occurred; this is based on previous same-day, test–retest (coffee-break) studies examining metastatic nodules. Whether results from prior studies apply to small non-metastatic nodules is unknown. This study aimed to establish the interscan variability in the volumetric measurements of small-sized non-metastatic nodules.

**Methods:**

Institutional review board approval was obtained for this study. Between March 2019 and January 2021, 45 adults (25 males; mean age 65 years, range 37–84 years) with previously identified pulmonary nodules (30–150 mm^3^) requiring surveillance, without a known primary tumour, underwent two same-day CT scans. Non-calcified solid nodules were measured using commercial volumetry software, and interscan variability of volume measurements was assessed using a Bland–Altman method and limits of agreement.

**Results:**

One hundred nodules (range 28–170 mm^3^; mean 81.1 mm^3^) were analysed. The lower and upper limits of agreement for the absolute volume difference between the two scans were − 14.2 mm^3^ and 12.0 mm^3^ respectively (mean difference 1.09 mm^3^, range − 33–12 mm^3^). The lower and upper limits of agreement for relative volume difference were − 16.4% and 14.6% respectively (mean difference 0.90%, range − 24.1–32.8%).

**Conclusions:**

The interscan volume variability in this cohort of small non-metastatic nodules was smaller than that in previous studies involving lung metastases of varying sizes. An increase of 15% in nodule volume on sequential CT may represent true growth, and closer surveillance of these nodules may be warranted.

**Key Points:**

*• In current pulmonary nodule management guidelines, a threshold of 25% increase in volume is required to determine that true growth of a pulmonary nodule has occurred.*

*• This test–retest (coffee break) study has demonstrated that a smaller threshold of 15% increase in volume may represent true growth in small non-metastatic nodules.*

*• Closer surveillance of some small nodules growing 15–25% over a short interval may be appropriate.*

## Introduction

Lung nodule growth is defined as an increase in nodule diameter or volume on sequential computed tomography (CT) scans and is a powerful predictor of lung malignancy [1, 2]. Measuring nodule growth can be performed using electronic callipers or semi-automated tools. However, establishing that true nodule growth has occurred can be challenging in cases where the increase in nodule size is small, due to the inherent limitations of measurement tools [3]. For this reason, many clinical practice guidelines stipulate that a minimum increase in nodule size must be achieved before nodule growth can be determined. For example, the Fleischner Society guidelines stipulate that a threshold of 2-mm diameter growth should be used to define true nodule growth [4]. By contrast, the British Thoracic Society (BTS) nodule management guidelines stipulate a 25% increase in nodule volume to determine growth [5], a threshold also used in the Nederlands–Leuvens Longkanker Screenings Onderzoek (NELSON) lung cancer screening trial [6, 7].

The threshold of a 25% increase in volume to determine true nodule growth in the BTS guidelines and NELSON nodule management protocol was based on a number of in vivo “coffee break” studies, in which individuals were scanned twice on the same day, demonstrating up to 25% variability in nodule volumes [8–10]. This variability has been attributed to slight differences in nodule segmentation, including for nodules which are irregular in shape, or attached to pleural surfaces or vessels [9], and to patient factors, such as the level of patient inspiration or pulsation of the heart [10].

However, results from previous so-called coffee break studies may not necessarily be applicable to smaller nodules detected incidentally or in lung cancer screening. This is because prior studies have mainly involved participants with known lung metastases ranging in size from < 10 to 5000 mm^3^. Lung metastases are known to be more likely to be smooth in outline and spherical in shape than incidentally or screen-detected nodules, and the interscan variability of such nodules has previously been demonstrated to be less than irregular and non-spherical nodules [8]. Furthermore, small nodules might be expected to show greater variability in nodule volumes than larger nodules due to their greater surface area-to-volume ratio [3].

The purpose of this study was therefore to determine the interscan variability in nodule volume measurements and to establish whether the 25% threshold to determine true growth [5, 6] was applicable in the setting of small non-metastatic pulmonary nodules.

## Materials and methods

Institutional review board approval was obtained for this study. Written consent was obtained from all recruited participants.

Seventy adults who were due to attend a scheduled clinically required follow-up at the Royal Brompton Hospital for either screen- or incidentally detected non-metastatic, solid, non-calcified, small-sized lung nodules < 150 mm^3^ between March 2019 and January 2021 were invited by letter to participate in this study. Written informed consent was obtained from forty-nine adults who agreed to participate in this study.

All 49 participants underwent an initial low-dose CT (LDCT) scan (scan 1) for clinical follow-up. Scans were immediately reviewed by a thoracic radiologist (with 3 years of experience in subspecialty thoracic radiology) to assess for the presence of the known lung nodule. In four participants, the nodule previously identified had resolved (*n* = 2) or appreciably reduced in size to < 30 mm^3^ (*n* = 2); these participants were therefore not recruited to the study, leaving 45 participants (25 males, median age 65 years, range 37–84 years) in the final study cohort. These individuals left the CT table after the first scan (scan 1), and subsequently during the same appointment were repositioned on the CT table, and scanned a second time (scan 2) after a short interval (2–13 min) using identical scanning parameters.

### CT image acquisition and transfer

All participants were scanned on a 128-slice Siemens Somatom Definition Edge multidetector CT scanner (Siemens Healthineers). Participants were scanned in a supine position from the lung apices to the lung bases, with a breath hold, and without the administration of contrast medium. Weight-based scanning parameters were used such that tube voltage was pre-set and selected for patient weight (< 50 kg at 100 kV, 50– < 80 kg at 120 kV, and ≥ 80 kg at 140 kV). Automatic exposure control (Care Dose4D) was enabled, resulting in adaptation of the tube current (mAs) based on patient size. Scanning was performed with a gantry rotation time of 500 ms. Images were reconstructed using filtered back projection with a soft tissue reconstruction kernel (B30f) [11], with a slice thickness/increment of 1 mm/0.7 mm. Images were transferred to the local PACS system (Impax, Agfa Healthcare).

### CT reading

Semi-automated nodule volumetric analysis was performed using SyngoVia™ software (Syngo.Via Client 5.1, SyngoVia™, Siemens Healthineers). Images were displayed in the SyngoVia™ software with a window width of 1500HU and level of − 500 HU. To assess interscan variability, all CT reading was performed by one radiologist (E.B.). Nodules were selected by drawing a line across the nodule using a mouse and were automatically segmented and measured by the software. No manual correction of volumetric measurements was performed; if segmentation was judged to be poor, for example because of attachment to an adjacent vessel or the pleura, nodules were excluded from analysis. Saved images showing measured nodule volumes were sent to the local PACS system. Nodules were characterised as being spherical or non-spherical. Non-spherical nodules were further sub-categorised as having a smooth or an irregular margin. All nodules in the range 30–150 mm^3^ were included in this study. Nodules smaller than 30 mm^3^ were excluded from analysis, as such nodules would not ordinarily warrant follow-up in clinical practice or in the screening setting after either a baseline or incident round scan. Larger nodules > 150 mm^3^ are approaching a size threshold for intervention or further investigation (> 200 mm^3^) in several screening protocols and have also been studied extensively previously, and thus were not evaluated.

As a secondary analysis, inter-observer variability in nodule volume measurements was assessed. The recorded nodules volumes for the first observer (E.B.) were deleted from the Syngovia™ volumetry platform. Following this, a second observer, with 3 years thoracic radiology experience (B.R.), independently re-measured and recorded the nodule volumes on the first of the two scans performed, blinded to the measurements of the first observer. If a nodule was felt to be poorly segmented by the software by the second observer, this was also recorded.

### Statistical evaluation

The inter-scan variability of volumetric nodule measurements was assessed using the Bland–Altman method of assessing agreement [12, 13]. Since the true volume of any given nodule is not known, the mean of the two measured volumes (*V*_1_ − *V*_2_/2, *V*_mean_) is assumed to represent the true nodule volume. In line with previous studies, the absolute difference in the measured nodule volumes (*V*_1_ − *V*_2_, mm^3^) is plotted against the mean nodule volume [8, 10, 14–16]. The difference in nodule volume is also shown as a proportion of the mean nodule volume ((*V*_1_ − *V*_2_/*V*_mean_) × 100, %), to provide a measure of the relative difference in volumes between the two scans. The upper and lower limits of agreement are calculated as the range of 95% of the observed absolute and relative differences in the two volume measurements (1.96 standard deviations above and below the mean difference). A sub-analysis was also performed of the limits of agreement for the smallest nodules, 30– < 80 mm^3^, compared to larger nodules 80–150 mm^3^.

To assess interobserver variability of nodule measurements, the same methods were used to establish the upper and lower limits of agreement in the absolute and relative volume measurements of the 100 nodules measured by both observers.

All statistics were carried out in SPSS version 26 and 28 (IBM SPSS Statistics for Windows).

## Results

**Fig. 1:**
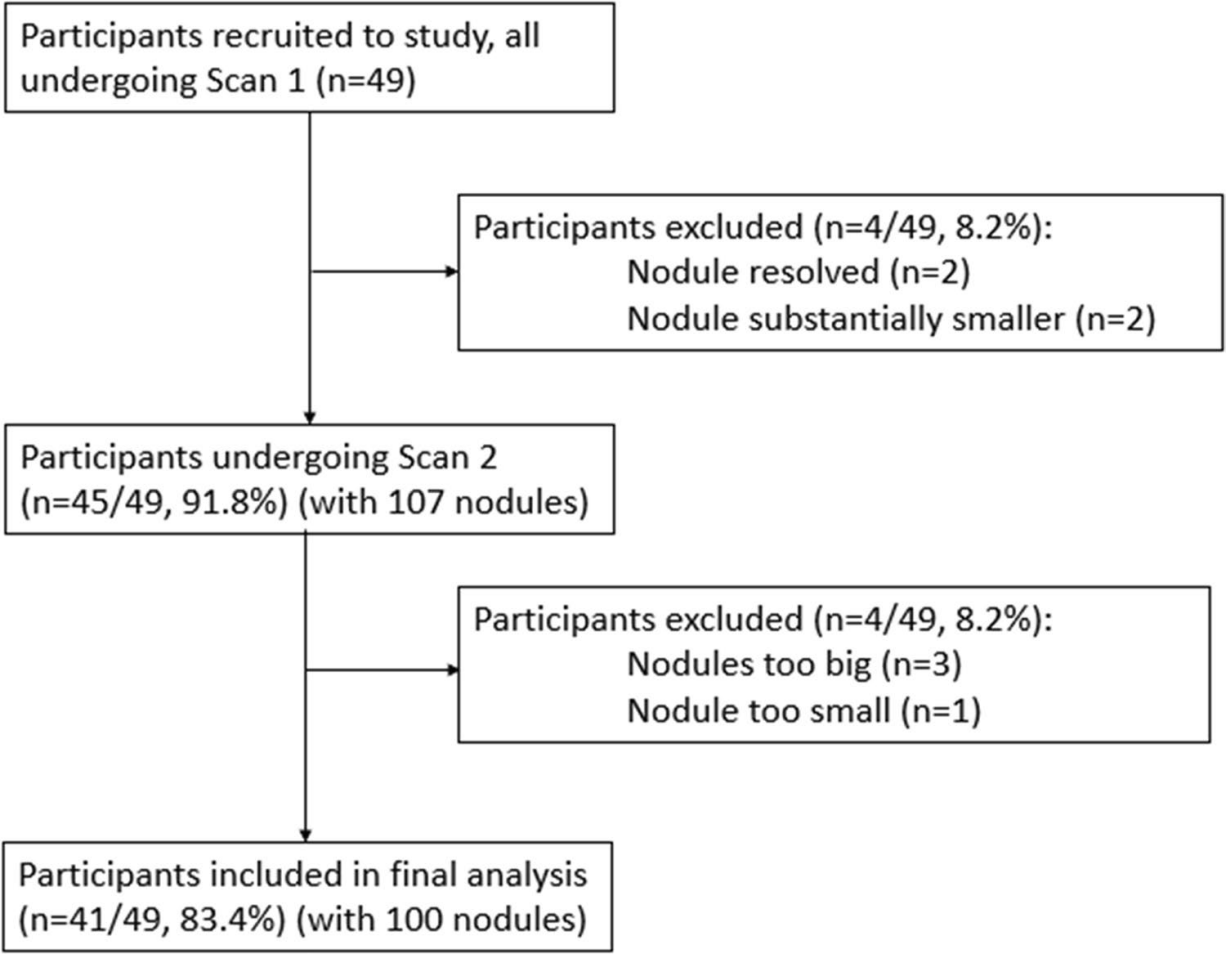
Flow diagram of participant inclusion

A total of 107 small-sized nodules were identified in 45 participants undergoing two LDCT scans. Four nodules were > 150 mm^3^ on the volumetric measurements on both the initial and second scans, and one nodule was just under 30 mm^3^ on both scans. The first observer recorded that two nodules were poorly segmented by the volumetry software; one was irregular in contour and juxtavascular, and the second nodule was irregular but not abutting the vasculature or pleura. All seven nodules were excluded from analysis. Therefore, a total of 100 non-calcified nodules in 41 participants were evaluated (Fig. 1). The demographic characteristics of the 41 study participants in whom nodules were finally evaluated, as well as the characteristics of the nodules examined, are given in Table 1.

**Table 1:** Demographic characteristics of 41 participants included in final analysis, with nodule characteristics of 100 evaluated nodules

Study participants—demographics	
Age (years) (median, range)	67.2 (37–84)
Sex (no. of patients, %)	
Male	23 (56.1%)
Female	18 (43.9%)
Nodule characteristics	
Total number of nodules	100
Lobe (no. of nodules, %)	
Right upper lobe	26 (26%)
Right middle lobe	6 (6%)
Right lower lobe	21 (21%)
Left upper lobe	21 (21%)
Left lower lobe	26 (26%)
Nodule morphology (no. of nodules, %)	
Spherical, smooth margin	28 (28%)
Non-spherical/polygonal, smooth margin	7 (7%)
Non-spherical, irregular margin	65 (65%)
Nodule location	
Freestanding intraparenchymal nodules	80 (80%)
Juxtapleural nodules	2 (2%)
Perifissural nodules	5 (5%)
Juxtavascular nodules	13 (13%)

**Fig. 2:**
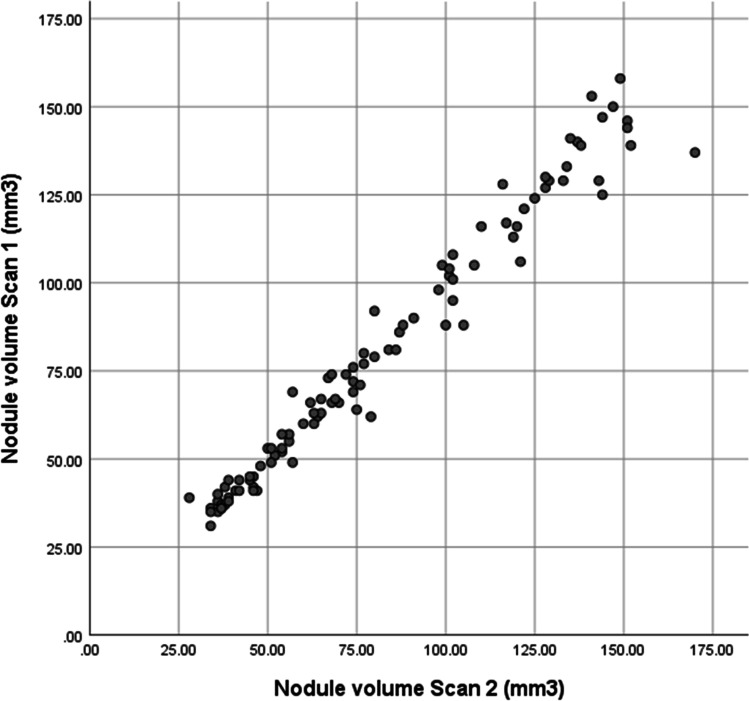
Volumetric measurements of 100 nodules on scans 1 and 2

 Nodule volumes on scan 1 ranged from 31 to 158 mm^3^ (mean 80.5 mm^3^, SD 36.5 mm^3^), and on scan 2 from 28 to 170 mm^3^ (mean 81.6 mm^3^, SD 37.6 mm^3^) (Fig. 2).

**Fig. 3:**
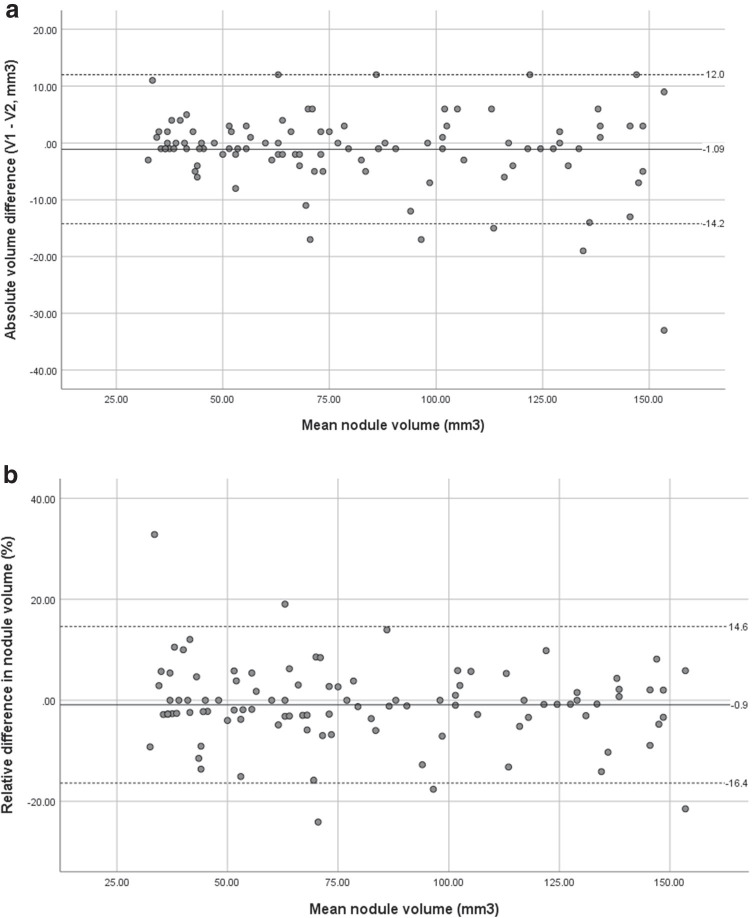
**a** Absolute volume difference (mm^3^) between volume measurements on the first and second scans, plotted against the mean nodule volume (mm^3^). Solid line demonstrates the mean absolute volume difference, and dashed lines show the upper and lower limits of agreement. **b** Relative difference in nodule volumes (%) plotted against the mean nodule volume (mm^3^). The solid line demonstrates the mean relative volume difference, and dashed lines show the upper and lower limits of agreement

The mean absolute volume difference between the two scans was − 1.09 mm^3^ (95% confidence interval − 2.42 to 0.24), range − 33 to 12 mm^3^. The lower and upper limits of agreement were − 14.2 mm^3^ and 12.0 mm^3^ (Fig. 3a). The mean relative difference in nodule volumes was − 0.90%, range − 24.1 to 32.8%. The lower and upper limits of agreement were − 16.4 and 14.6% respectively (Fig. 3b). Lower and upper limits of agreement for the relative difference in nodule volumes for 58 nodules with a mean volume of 30– < 80 mm^3^ were − 16.8 to 16.2%, with a mean relative volume difference of − 0.3% (95% confidence interval − 2.51 to 1.91%). For the 42 larger nodules, 80–150 mm^3^, the upper and lower limits of agreement for the relative difference in nodule volumes were − 15.8 to 12.3%, with a mean relative volume difference of − 1.7% (95% confidence interval − 3.96 to 0.50%).

Identical nodule measurements were made by the second observer in 95/100 nodules measured. The second observer determined that one additional nodule was poorly segmented by the volumetry software, but otherwise agreed with the first observer, that the remaining 99 nodules measured were well segmented. The mean absolute volume difference between the measurements of the two observers of the 100 nodules was − 0.39 mm^3^ (95% confidence interval − 0.85 to 0.73), range − 20 to 2 mm^3^. The lower and upper limits of agreement were − 4.96 to 4.18 mm^3^ respectively. The mean relative volume difference between the measurements of the two observers was − 0.03% (95% confidence interval − 0.38 to 0.43), range − 12.1 to 14.8%. The lower and upper limits of agreement for the relative difference in nodule volumes measured by the two observers were − 3.98 to 4.01%.

In view of the wider limits of agreement for the smaller nodules < 80 mm^3^ than the larger nodules 80–150 mm^3^, a post hoc analysis was performed using linear regression to assess for proportional bias. The values of the relative difference in nodule volumes (%) were converted to absolute values (removing negative signs) and regressed against the mean nodule volume [17]. This demonstrated no significant relationship between the relative difference in nodule volume and the mean nodule volume (*p* = 0.66).

Current nodule management guidelines assume that an increase in volume of < 25% represents stability [5, 7, 18]. Given that we found that the relative volume difference was approximately 15.5% either side of the mean nodule volume, and therefore that any increase above this may represent true growth, we performed a further post hoc analysis to model how these results impact current nodule management guidelines for small nodules (Table 2). Specifically, we modelled the impact of assuming that an increase of 15–24% represented true growth. Furthermore, we assumed that the optimal threshold for intervention and further investigation in growing small nodules was 200 mm^3^ [18–21]. As shown in Table 2, the assumption of nodule stability if a nodule is growing < 25% but > 15% between serial scans may results in a short delay to further investigation for some small nodules.

**Table 2:** A model to demonstrate the implications for management of solid nodules, assuming that an increase in volume of 15–24% in 3 months represents true growth, compared to current guidelines [5, 18]. *VDT* volume doubling time

Nodule volume (mm^3^) at baseline	Modelled nodule volume range at 3 months assuming 15–24% increase in size (mm^3^)	Nodule management as determined by current guidelines which assume that a < 25% increase represents stability	Modelled nodule volume range (mm^3^) at 12 months assuming consistent growth (VDT 296–456 days^a^)	Optimal time at which intervention would be warranted^b^ assuming consistent growth	Implication for current nodule management assuming 15–24% increase in volume represents genuine growth
30	34.5–37.2	Assumed stable; perform 12-m scan	52.5–70.9	27–42 months	No change in management at 12 m
50	57.5–99.2	Assumed stable; perform 12-m scan	87.5–118.2	21–30 months	No change in management at 12 m
80	92–99.2	Assumed stable; perform 12-m scan	139.9–189.1	15–21 months	No change in management at 12 m
115	132.3–142.6	Assumed stable; perform 12-m scan	201.1–271.9	9–12 months	Up to 3 m delay in investigation^c^
130	149.5–161.2	Assumed stable; perform 12-m scan	227.4–307.3	6–12 months	Up to 6 m delay in investigation^d^
150	172.5–186	Assumed stable; perform 12-m scan	262.3–354.6	6–9 months	Up to 6 m delay in investigation^d^

^a^A VDT of 296–456 days corresponds to between 15 and 24% growth over a period of 3 months

^b^This assumes intervention occurs in growing nodules when 200 mm^3^ in size[18–21]

^c^In this case, the nodule in question would reach 200 mm^3^ at between 9 and 12 months of follow-up, therefore resulting in a delay of up to 3 months to investigation

^d^In these cases, the nodule in question would reach 200 mm^3^ at between 6 and 12 months of follow-up, therefore resulting in a delay of up to 6 months to investigation

## Discussion

This study sought to establish the reproducibility of volumetric software in measuring small non-metastatic pulmonary nodules < 150 mm^3^, using established methods. Given the effects of partial voluming on CT, and the non-spherical nature of many nodules detected incidentally in the clinical and lung screening setting, the limits of agreement were expected to be wider than published in previous similar studies involving metastatic nodules. Unexpectedly, this study demonstrated narrower limits of agreement, 15.5% either side of the mean relative difference in nodule volume. The upper and lower limits of agreement for the relative difference in nodule volumes measured by two observers were only ± 4%, confirming data from previous studies that interobserver variability in nodule volume measurements contributes very little towards the overall variability in nodule measurements [9, 10].

Compared to previous similar studies where the morphological characteristics of nodules have been compared [8, 10], a greater proportion of the nodules in the current study were irregular in contour, as was predicted due to the non-metastatic nature of nodules included in this study. Despite this, the current study has shown narrower limits of agreement for the relative nodule volume measurements than the majority of previous studies [8–10, 15, 16]. This may reflect advances in volumetry software over the past 15 years, differences in volumetry software packages, and advances in CT scanner technology. One study by Zhao et al demonstrated narrower 95% limits of agreement (− 12.1 to + 13.4%) in the volumetric measurements of a cohort of 32 known non-small-cell lung cancers; however, the mean tumour size in this study was > 3-cm diameter, and results are therefore not directly comparable to the current study of very small lung nodules [22]. The impact of nodule size on measurement variability has been studied in a small number of previous in vivo studies with conflicting results [8–10, 15]. Wormanns et al found very similar limits of agreement in nodules ≤ 10 mm in diameter, compared to those > 10 mm in diameter [9], whereas Goodman et al found that confidence limits narrow with increasing nodule volume [10]. Comparing the volumetric measurements of metastatic nodules < 8 mm and ≥ 8 mm in diameter, De Hoop et al found that the interscan variability in nodule volumes decreased with increasing nodule volume when measured by 5 of 6 nodule volumetry software packages [16]. By contrast, Talwar and colleagues found lower variability for nodule measurements in nodules < 500 mm^3^ compared to those over 500 mm^3^ [15]. However, no previous studies have focussed specifically on small nodules < 150 mm^3^ (equivalent to < 6.59-mm diameter), frequently encountered in both the lung cancer screening setting, and incidentally on CT scans of the chest. This study demonstrated no evidence that the relative difference in nodule volumes (%) varies in relation to the absolute size of the nodule for small nodules between 30 and 150 mm^3^.

A number of nodule management guidelines in the clinical and screening setting recommend a 3- or 6-month follow-up CT for solid nodules in the size range 30–150 mm^3^ [5, 7, 19, 20], the lower limit applying to new nodules developing on incident round screening CTs [23]. Results from this study indicate that > 15% growth in a nodule volume may represent true nodule growth in this cohort of small nodules, and that an assumption of stability should not be made for nodules growing 15–25% over 3–6 months. This assumption of stability may result in a short delay to investigation of a cohort of nodules measuring 115–150 mm^3^, and these may require closer short-term surveillance, for example, with a repeat scan in a further 3–6 months, particularly as nodules approach a threshold for intervention (> 200 mm^3^). This would prevent a potential delay to diagnosis of malignancy in growing nodules in this size range. Provided that a minimum volume threshold of 200 mm^3^ is maintained with nodule management protocols prior to intervention, little harm is expected to result from lowering the threshold to determine growth to 15% in this cohort of nodules 115–150 mm^3^, since at least one further short-term interval scan would be required to confirm persistent growth prior to any invasive procedure. This study did not evaluate nodules in the size range 150–300 mm^3^ which are also considered indeterminate in a number of nodule management protocols utilised in screening [5, 18–20, 24]. As several previous studies have incorporated larger nodules and found wider limits of agreement (around ± 25%), maintaining the threshold of 25% growth to confirm true growth may be appropriate for larger nodules to prevent potential over-investigation of nodules in the range 150–300 mm^3^. Further studies are warranted in this regard.

Strengths of the study include that all individuals were scanned with an identical low-dose scanning protocol on the same scanner, and analysis was performed using a modern volumetry software package. Therefore, many of the conditions in which this study was performed are likely to closely mirror conditions in a lung screening cohort. A limitation of the current study is that the results have been obtained using a single software package and therefore may not apply to other volumetry packages. Studies of similar patient cohorts using other software packages are warranted. It is noted that, in clinical practice, further variability may be introduced through several other variables including CT acquisition factors (such as dose), reconstruction techniques (filtered back projection or iterative reconstruction), and reconstruction parameters including slice thickness. Of these factors, reconstruction algorithm and slice thickness are the primary contributors to interscan variability [3]. As much as possible, all such parameters must be kept constant between scans comparing nodule volumes. In the event that scan acquisition and reconstruction parameters are not constant between scans, it may be more appropriate to use the 25% threshold for growth currently in use. However, in the screening setting, scans are ordinarily performed with identical scanning parameters and therefore the findings of our current study would apply in this context. It is also noted that the findings of this study should not be extrapolated to nodules which are poorly segmented by volumetry software and therefore measured by diameter. Such nodules may require ongoing surveillance for an extended period (up to 2 years) based on nodule diameter.

In conclusion, this study has demonstrated that, for small non-metastatic pulmonary nodules, true growth can be reliably concluded to have occurred with a volume change of > 15% where scanning parameters are identical between scans. Caution, therefore, should be exercised in participants with nodules growing 15–25%, particularly those nodules in the range of 115–150 mm^3^. Under current nodule management guidelines widely used in the clinical and screening setting, such nodules would be presumed to be stable, and closer surveillance of these nodules may be warranted.

## Abbreviations

BTS: British Thoracic Society
CT: Computed tomography
LDCT: Low-dose computed tomography
NELSON: Nederlands–Leuvens Longkanker Screenings Onderzoek (Trial)
PACS: Picture Archiving and Communication System
V: Volume
VDT: Volume doubling time

## Declarations

### Guarantor

The scientific guarantor of this publication is Professor Anand Devaraj.

### Statistics and biometry

No complex statistical methods were necessary for this paper.

### Methodology

• prospective

• performed at one institution
